# Using Quantitative Passive Thermography and Modified Paris-Law for Probabilistic Calculation of the Fatigue Damage Development in a CFRP-Aluminum Hybrid Joint

**DOI:** 10.3390/polym13030349

**Published:** 2021-01-22

**Authors:** Jannik Summa, Hans-Georg Herrmann

**Affiliations:** 1 Chair for Lightweight Systems, Saarland University, Campus E3.1, 66123 Saarbrücken, Germany; hans-georg.herrmann@izfp.fraunhofer.de; 2 Fraunhofer Institute for Non-Destructive Testing (IZFP), Campus E3.1, 66123 Saarbrücken, Germany

**Keywords:** CFRP-hybrid, fatigue crack growth, non-destructive testing, quantitative evaluation

## Abstract

Although metal to Carbon-fiber-reinforced-polymer (CFRP) hybrid-joints possess a high lightweight construction potential, their extensive application has to deal with interfacial stress concentrations promoting fatigue damage. Furthermore, the underlying damage processes and their influencing factors are still not completely understood. Besides interfacial property-gradients, generic shapes counteract a precise determination of local stresses or strains, respectively. Hence, new methods are required that combine non-destructive testing and fracture mechanics to account for the fatigue damage. In this work, data of mechanical fatigue testing of an aluminum-CFRP hybrid-structure is presented by means of the dynamic stiffness and the mechanical hysteresis. Additionally, in situ passive thermography allows for capturing the heat development due to delamination growth. Correlating the obtained data implies that faster delamination growth coincides with higher amplitude values of lock-in thermography and higher mechanical hysteresis. Supported by this observation, a model is formulated to calculate the local dissipation per loading cycle. Further integration into a Paris-law like formulation results in a calculation model to account for the mode-I fatigue delamination growth. Additional validation of the model parameters shows good agreement with the experimental data.

## 1. Introduction

With increasing relevance of multi material hybrids, e.g., metal to carbon-fiber-reinforced-polymer (CFRP) hybrids, knowledge about their lifecycle performance and evaluating their condition under mechanical fatigue became a major issue. As the characteristic damage mechanisms and the crack resistance depend as well on the constituents as on their joint, the relevant state of the art research concerns both, the CFRP itself and hybrid-joints.

For CFRP laminae fundamental research has been carried out based on well-defined sample geometries, facilitating precise stress calculations and giving criteria for crack growth related to a physical or continuum-mechanical background. In contrast, the generic geometry of most metal-CFRP hybrids and the unknown effect of interfacial traction-forces impede exact measurements of stresses and strains at critical locations, i.e., the interfacial region or interlaminar stresses. Not least of all, production-induced and inherent statistical fluctuations of material properties increase the addressed uncertainties. Therefore, a variety of application related research appeared in the recent decades, contributing to the understanding of hybrid-structures by characterizing the complex interaction of influencing factors, with some simplifications as an inevitable compromise. Thus, giving helpful tools at hand, which enable us to estimate long-term behavior and dimensioning. 

However, for characterization purposes, thermography is one of the most promising non-destructive testing techniques (ndt). It captures the thermal effects that occur due to damage and crack growth, as well as periodically varying stresses [1]. Thus, facilitating the correlation of the location-dependent amount of temperature-variation with macroscopic mechanical measurands [2], it provides the possibility of contemplating inhomogeneous stress states in hybrid-joints. This procedure can be found in the state-of-the-art literature for determination of stress-distribution [3] and mainly for qualitative detection of defects [4], whereas damage assessment methods ask for new approaches crosslinking quantitative NDT information and fracture mechanics.

This work aims to use passive thermography as a characterization method that results in quantitative caloric information, which serves as an input for energy-based principles of fracture mechanics. Therefore, it is reasonable to contemplate related topics, such as fracture models for CFRP and hybrid-joints, damage in hybrid-joints and quantitative thermography.

### 1.1. Literature Review on Damage in CFRP

In order to evaluate the life-cycle performance of CFRPs different criteria have been developed, i.e., energy and damage-tensor based as well as fracture mechanical models [5]. The damage-tensor approach was mostly applied, when major attention was paid to the macroscopic behavior [6,7]. A more differentiated view is given by [8], summarizing the various failure mechanisms in composite materials with corresponding estimations of the failure-energy. Specific fracture energies were also cited for transverse fiber fracture, 20–60 kJ/m^2^, and delamination, 100–3000 J/m^2^ depending on matrix material and strain rate, respectively. 

Furthermore, the failure behavior depends on the relation of fiber-matrix adhesion strength and the scattering of fiber strength [9]. Talreja [10] assigns these mechanisms to particular regimes of fatigue failure, i.e., short cycle or long cycle fatigue. However, applying the equivalent initial flaw size approach [11] or integrating NDT techniques can enhance the assessment of the life-cycle performance [8,12,13].

Since mode-I and mode-II stresses are detrimental to the laminate and lead to delamination, special attention is paid to them. Under static loading conditions the crack growth can be described based on the characteristic critical strain energy release rate (GIC). It can be derived based on the beam theory [14,15], while its value depends on the crack-length and the strain rate [15]. Furthermore, there are different formulations to account for mode-I and mode-II fatigue delamination growth [16], while most of them are empirical or Paris-law like formulations and refer to the critical energy release rate.

The fatigue behavior and propagation of damage of cross-ply CFRP under various in-plane loading conditions have been studied by Skinner et al. [17]. Multi-scale studies of fracture surfaces with electron microscope and light microscope and measurements of the specimens’ stiffness degradation gave an insight into the initiation and propagation of damage mechanisms. Contrary to experimental studies, Deng et al. [18] have proposed a sophisticated failure analysis framework to predict intralaminar failure and composite strength under multiaxial loads. 

### 1.2. Literature Review on Damage in Hybrid-Joints

In most of the recent works [19,20,21], the hybrid-joint was manufactured by adhesives, screws or pins. Hence, direct contact prevailed between the metal and the CFRP constituent, resulting in a mechanical gradient at the interface. Consequently, bonding and mechanical gradients at the interface play a major role for the mechanical properties of the joint. Moreover many of these works reported a crack promoting effect due to the mechanical gradient and due to interpenetration of the constituents [22,23,24]. Ply drops, bond joints, free edges and notch holes generate interlaminar stresses which promote delamination [16].

To counteract mechanical stress concentrations in the bonding region, Pohl et al. [25] developed a hybrid-structure with a thermoplastic constituent between the aluminum and the CFRP constituent for interfacial stress relaxation. Investigations into the fatigue crack growth have shown that the properties of the constituent in the bonding region is crucial [26]. In situ passive thermography investigations have shown that delaminations are the dominant damage mechanisms, if medium to soft thermoplastic material is present in the joining zone, wherein translaminar cracking leads to failure in the presence of aluminum.

### 1.3. Literature Review on Thermography for Damage Detection

Regarding non-destructive testing as a damage characterization technique, thermography was often applied under quasi-static [3,4,27] and fatigue loading [28]. Thereby, convenient thermal properties of CFRP have led to thermography signal mapping, with hot spots indicating the failure location [27,29,30]. Various works reported on the unique relation between the cyclically introduced mechanical energy and the thermo-elasticity [31,32] or heat build-up [2,33], respectively. 

A detailed analysis of the thermography signal can be found in [1]. According to their work, the thermal signal consists of the mean temperature and a periodic term, which represents the short-term response to mechanical excitation, i.e., thermoelastic signal. Due to the periodic nature of the short-term response, they state, the lock-in transformation of the thermal signal results in the best damage detectability and may even improve the accuracy of failure predictions. While leaning their predictions on the thermal phase [1], many works measured the heat-build up during fatigue experiments for failure predictions [2,28,34]. According to Steinberger et al. [2] the heat build-up is directly correlated with the dissipated thermal energy, which is confirmed by experimental results given in [33]. They continue that the increase of the dissipated energy can be fitted with an exponential fit. Thus, giving an empirical equation incorporating the lifetime to failure and the dissipated energy.

However, both effects, thermo-elastic heating as well as dissipative heating, contribute to the overall change in temperature. It can be described with the local heat-conduction equation, which is derived from the Clausius-Duhem inequality (see Equations (1) and (2), [35]). It relates the overall time derivative of temperature with the intrinsic (mechanical) dissipation *d*_1_, thermoelasticity, heat loss and transitions of thermodynamic states *α* and strain *ε*:

(1) $$\rho C_{\varepsilon ,\alpha }\dot{T}+div\;q=d_{1}+\rho T\psi_{,T,\varepsilon }:\dot{\varepsilon }+\rho T\psi_{,T,\alpha }\cdot \dot{\alpha }+r_{e}$$

(2) $$d_{1}=\sigma :D-\rho \psi_{,\varepsilon }:\dot{\varepsilon }-\;\rho \psi_{,\alpha }\cdot \dot{\alpha }\geq 0$$

With density *ρ*, specific heat capacity *C_ε,α_*, intrinsic dissipation *d*_1_, derivation of temperature with respect to time $\dot{T}$, *r_e_*: external heat source, *div q*: heat loss to the surroundings and Helmholtz free Enthalpy *Ψ* and its derivations with respect to the variables *i* $\Psi_{,i}$. The intrinsic dissipation *d*_1_ denotes the difference of anelastic and stored energy rate with Cauchy stress tensor *σ* and Eulerian strain rate tensor *D*.

Taking into consideration some simplifications, i.e., no external heat supply (*r_e_* = 0), the temperature variation (below 10 °C) being low enough to neglect the couplings between temperature and internal variables other than elastic variables (third term on right hand side of (1)), neglecting the variation of *ρ* and *C* and identifying the second term on the right hand side of (1) as the thermoelastic effect [34] leads to:

(3) $$\rho C_{\varepsilon ,\alpha }\dot{T}+div\;q=d_{1}+\rho Tk:{\dot{\varepsilon }}_{}$$

With density *ρ*, specific heat capacity *C_ε,α_*, intrinsic dissipation *d*_1_, derivation of temperature with respect to time $\dot{T}$, $\dot{\varepsilon }$: strain rate tensor and k the thermoelastic tensor. The latter is assumed to be isotropic for simplification.

Their approach [34] concludes in an equation that relates the cycles to failure with the increased temperature. Thus, giving a formula to estimate the lifetime to failure on a physical basis. However, Equation (3) represents the basis for the quantitative evaluation of thermography data and the new approach, which are discussed in Section 3.3.

## 2. Materials and Methods

**Figure 1:**
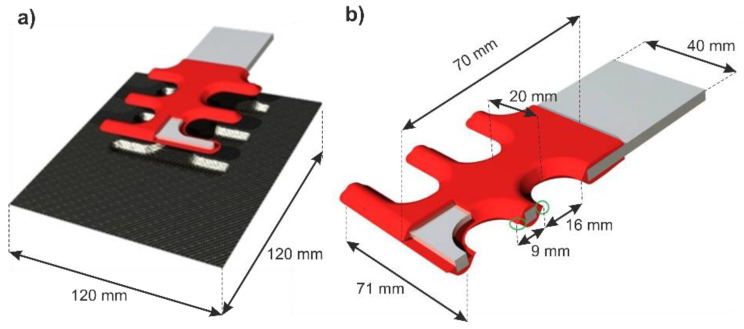
(**a**) Insert positioned between the CFRP-laminate, (**b**) geometric shape of the Al-Thermoplastic insert [25].

The investigated parts are manufactured by direct injection-overmolding of a 4 mm thick insert, Aluminum EN AW 6082 (AlMgSi1), with a 2 mm thick thermoplastic polymer. Subsequently, the overmolded insert is placed between the middle plies of the CFRP-laminate (see Figure 1) and consolidated in a resin transfer molding (RTM) process, thus resulting in a form fit; no further processing needs to be carried out. Note that according to Grujicic et al. [36] direct injection leads to mechanical interlocking on the micro-scale between the polymer and the aluminum-insert. The CFRP-Laminate consists of four 0.25 mm thick plies in a quasi-isotropic layup [0/90°, ±45°] _s_ with 30 vol% 3K plain weave (Torayca FT300B) carbon fiber and a two-component epoxy-system (Biresin CR170/CH150-3) as matrix. The CFRP-Laminate measures 120 mm in height, 120 mm in width and 1 mm in thickness. The pore content of the manufactured laminate was not measured.

In this work the experiments are carried out on hybrid-joints with two different thermoplastic materials. These are a highly rigid Polyphthalamid reinforced with 30 vol-% short glass-fibers (PPAGF30, Vestamid^®^ HT plus M1033, E-modulus: 11.2 GPa) and the same Polyphthalamid without reinforcement (PPA, Vestamid^®^ HT plus M1000, E-modulus: 3.5 GPa). In the latter, all specimens containing PPAGF30 are indicated as part of the ‘A-Series’ and the ones with PPA as part of the ‘B-Series’, whereas specimen-designations combine the material-reference (A or B) and a numeration, e.g., ‘B18’.

Additional experiments are conducted to determine the interlaminar tensile strength (*G_IC_*). Therefore, double cantilever beam specimens are produced according to DIN EN 6033 [37] with 150 mm length, 25 mm width and 2 mm thickness. The laminate is manufactured with the same material, but as an 8-ply laminate with [0/90°, ±45°, ±45°, 0/90°] _s_. Additionally, a piece of Teflon tape is placed between the middle plies to introduce a starting crack with length 9 mm. The *G_IC_* tests are driven with a cross-head displacement of 10 mm/min.

**Figure 2:**
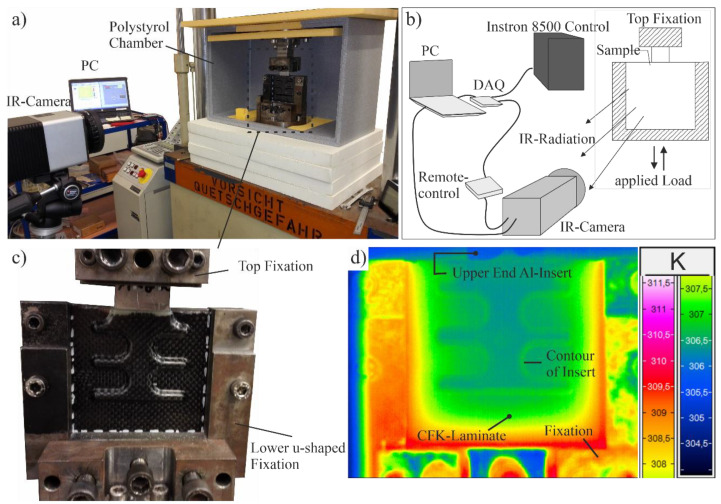
(**a**) Experimental setup with Instron 8500, thermography-camera and computer, (**b**) illustration of experimental setup with remote-control unit to synchronize IR-Camera and fatigue testing, (**c**) generic u-shaped fixation of specimens, (**d**) raw IR-image of specimen mounted in u-shaped fixation.

The mechanical tests are carried out on an Instron 8500 with a 100 kN load cell. The data acquisition rate is 1 kHz. For mounting the specimen side-screw action grips are used. The clamping length is 25 mm at the aluminum-insert and at the bottom of the CFRP-laminate, as well as 10 mm on left and right side of the CFRP-laminate (see Figure 2). The setup is complemented with a InfraTec VarioCam^®^ HD Head bolometer camera for in situ passive thermography. The camera has a resolution of 1024 × 768 pixels, a spectral sensitivity in the range of 7.5 to 14 µm and a noise equivalent thermal detectability (NETD) smaller than 0.05 K (at T = 303.15 K). The camera is placed at a distance of 70 cm orthonormal to the specimen adjusting the focus plane on the CFRP surface (Figure 2d).

The fatigue experiments are carried out load-controlled with a sinusoidal loading with a frequency of 5 Hz, R-ration (*F_min_*/*F_max_*) being 0.1 and the maximum applied force *F_max_* being 7.5 kN. The IR-camera is triggered at certain loading cycles *n* (every 100 cycles up to 10^3^ and every 5000 cycles), at each capturing 18 frames with 30 frames per second (fps). The fatigue experiments end when the specimens experience complete rupture.

## 3. Results

### 3.1. Fatigue Behavior and Damage Mechanisms 

**Figure 3:**
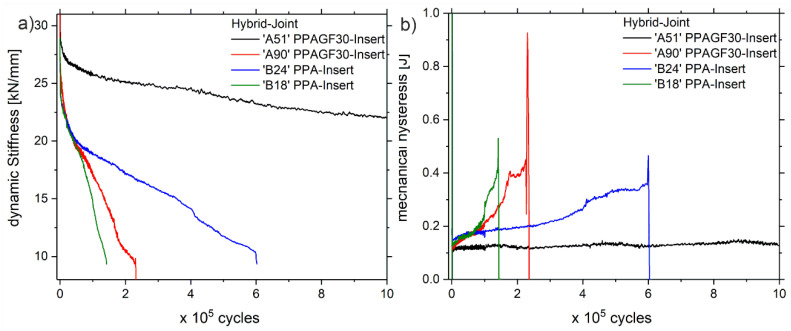
(**a**) Evolution of the dynamic stiffness of the hybrid-joints with PPA- and PPAGF30-insert-types during fatigue loading, (**b**) corresponding evolution of the mechanical hysteresis, note that two examples are displayed for each of the insert-types named ‘A51’, ‘A90’, ‘B24’ and ‘B18’.

A detailed and phenomenological discussion of the mechanical properties and damage development of the Al-CFRP hybrid joints under fatigue mechanical testing can be found in [26], from where the experimental data displayed in Figure 3 is adapted. Therefore, Section 3.1 is a brief summary of the phenomenological observations explaining the damage development and the accompanying thermal effects. Those are important in consideration of the following discussion on the new calculation model.

The dynamic stiffness (secant modulus of force over displacement) decreases successively due to fatigue mechanical loading, which is accompanied by an increase in the mechanical hysteresis (area under the closed force-displacement loop). Further, if the mechanical hysteresis is relatively high, the sample’s dynamic stiffness decreases faster. One possible explanation is that the dynamic stiffness decreases as the damage propagates, wherein a higher amount of energy is supplied for crack growth with a higher mechanical hysteresis. The data of sample ‘A51’ supports this hypothesis. The mechanical hysteresis is relatively low and the small decrease in dynamic stiffness fits to the small increase of the mechanical hysteresis from 0.11 J to 0.13 J.

**Figure 4:**
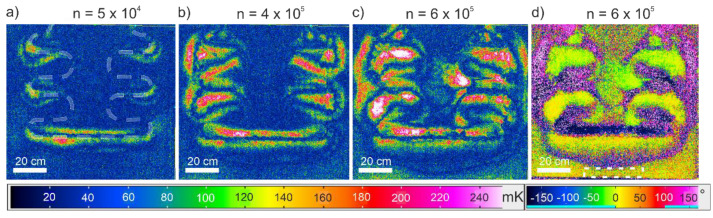
Lock-In amplitude images of sample ‘B24’ at (**a**) 5 × 10^4^, (**b**) 4 × 10^5^ and (**c**) 6 × 10^5^ loading cycles and (**d**) corresponding lock-in phase image with a dotted line indicating the reference area for the calculation model, (**a**) white dotted lines show the insert position.

The decrease of dynamic stiffness (and increase of mechanical hysteresis) is caused predominantly by the growth of delaminations, which originate under the insert and around the insert arms, subsequently propagating throughout the symmetry plane of the laminate (Figure 4). The process of delaminations growth is of a dissipative nature, thus leading to thermal dissipation in the process-zone. Due to the periodic nature of the mechanical excitation, the lock-in transformation of the IR-images results in a good contrast (see Figure 4). 

Each pixel of the displayed images represents the amplitude value of the 5 Hz sinusoidal change of the surface temperature within the measured time interval of 0.6 s (18 frames at 30 fps). Higher amplitude values (red to white colored) mean a higher change of temperature with respect to time, which means more dissipated heat. Amongst the lock-in amplitude images, a movement of the outermost yellow and red lines in Figure 4 (*T_amp_* values higher than 100 mK) is visible, which can be attributed to the movement of the crack front. Furthermore, it facilitates the measurement of the size of the delamination, while additional validation with x-ray computer-tomography is conducted (see [26]).

It is also evident from the lock-in amplitude images that the more the delaminations grow, the more areas with higher *T_amp_* values appear. Note that this goes hand in hand with the increase of the mechanical hysteresis (Figure 3b).

**Figure 5:**
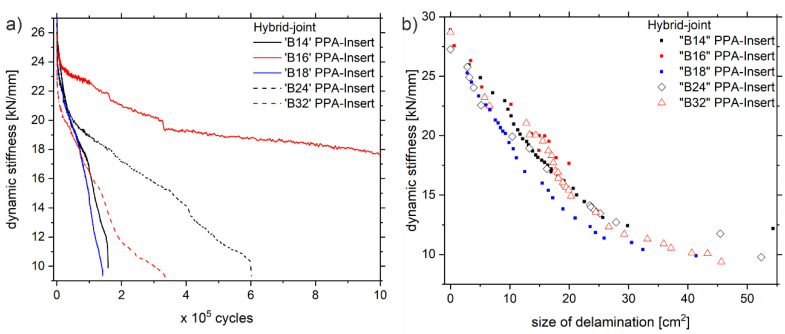
(**a**) Evolution of dynamic stiffness during fatigue loading with respect to loading cycles, (**b**) evolution of dynamic stiffness with respect to measured size of delamination.

The discussed evolution of delaminations is characteristic of all of the tested specimens of Series ‘A’ and ‘B’, even though a broad scattering is undoubtedly present (Figure 5a). Plotting the dynamic stiffness as a function of the delamination size reveals a strong dependency between these quantities (Figure 5b). Anyhow, the dependency between dynamic stiffness and delamination size does not reflect the testing-series’ scattering regarding the lifetime to failure or the crack growth rate, respectively, and thus is independent of time and rate.

Taking into consideration the discussion on Figure 5b and the hybrid-joint being designed as a form-fit, the authors assume that the residual dynamic stiffness, as well as the residual load bearing capability, is determined by the ratio of damaged and undamaged laminate. Consequently, the delamination size should be a viable quantity to indicate upcoming failure. 

**Figure 6:**
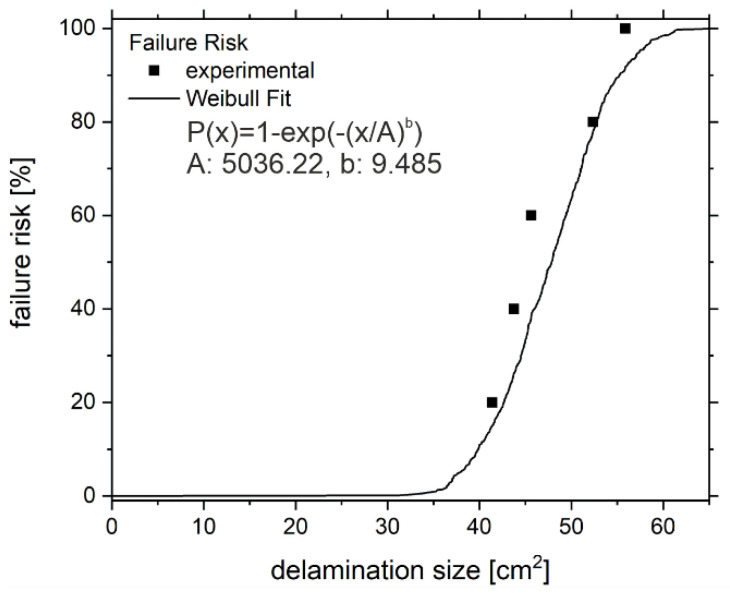
Failure risk as a function of the delamination size based on experimental data (B-series, black dots) and a Weibull-Fit (black line).

The analysis of the delamination size at the instance of failure for the specimens of the B-series is given in Figure 6. The cumulative amount of failed samples as a function of the delamination size can be expressed as the failure risk. In other words: with the sample failing at 56 cm^2^, all of the tested samples have failed resulting in a failure risk of 100%.

According to the experimental data, failure with a 50% risk can be expected above 41.3 cm^2^ and approaches 100% failure risk at 55.8 cm^2^. A fitting approximation can be found using the Weibull-plot with coefficients *A* = 5036.2 and *b* = 9.485 for the failure risk:

(4) $$P\left(x\right)=1-exp\left(-{\left(\frac{x}{A}\right)}^{b}\right)$$

However, the discussed results show that the delamination size is not sufficient to explain the scattering of the lifetime to failure, implying a missing explanation for the differences in the crack growth rate. So, to close this section with an important observatory remark, note that higher amplitude-values of lock-in thermography always coincide with a higher mechanical hysteresis, which means higher internal dissipation in the specimen. Additionally, specimens with a higher mechanical hysteresis are found to experience faster delamination growth. Hence, the lock-in amplitude is at least a qualitative indicator for the crack growth rate. 

### 3.2. Mode I Interlaminar Fracture

In a fracture mechanical perspective, the growth of delaminations in the laminate’s symmetry plane is described as mode I crack growth with the characteristic threshold parameter for crack growth being the critical strain energy release rate or interlaminar tensile strength *G_IC_*, respectively. Therefore, according to DIN EN ISO 6033:2015 [37] a standardized experiment was carried out on double cantilever beam specimen, which are produced with the same CFRP-fabric and matrix as the hybrid joint (see Section 2). Three samples were tested for each of the two batches (batch 3 and 4). Evaluation of the results uses the compliance-beam-theory [15]. The resulting values for the critical strain energy release rate *G_IC_* are displayed in Table 1. According to the results, the critical strain energy release rate amounts 486 (±84) J/m^2^.

### 3.3. Calculation Model for Delamination Growth Based on Thermography Data

Recall the Equations (1) and (3) on the local heat conduction equation, which relates the overall time derivative of the temperature with the intrinsic dissipation *d*_1_, thermo-elasticity, heat loss and the transition of thermodynamic state variables.

Since the intrinsic dissipation *d*_1_ can be seen as the caloric quantity being transferred from external mechanical energy into the system due to irreversible strains and damage, the part of temporal change in temperature that accounts for the dissipational processes is of major interest. 

**Figure 7:**
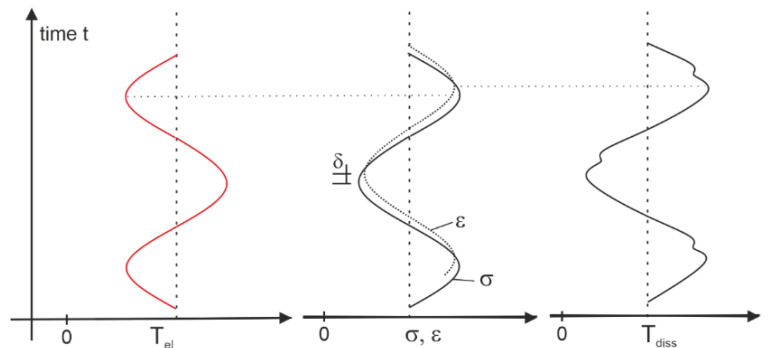
Externally applied mechanical stress ε causes the strain δ with inelastic delay *δ*, which leads to thermoelastic *T_el_* (left) and dissipational *T_diss_* (right) variations of temperature with a phase shift of approximately 180°.

The observable difference over time of thermoelastic and dissipational heating caused by externally applied cyclic loading is illustrated in Figure 7. Non-cracking regions are dominated by thermoelasticity, where the temperature increases as the load decreases. The opposite applies for regions of crack growth. Accordingly, the phase-shift between thermo-elastic and dissipational heating is approximately 180° or π respectively, if the material exhibits low viscoelasticity. This very phenomenon can be observed from the experimental results (Figure 4d). 

Subtracting the phase-offset, so the phase in the reference-area (area that remains un-damaged, see Figure 4d) equals 0°, results in areas within the phase-image displaying a phase-shift of approximately 180°. These areas agree perfectly with the relatively high lock-in values (red and white-colored in Figure 4c), which indicate the zones of crack propagation.

However, the dissipational and thermoelastic contributions can be separated from each other and calculated based on thermography measurands—namely the lock-in temperature-amplitude *T_amp_* and the phase-shift ϕ (see Figure 7). Note that the environmental loss, i.e., convection, radiation and conduction, is neglected, since one instance of thermography images covers the short observation period of 0.6 s (18 frames at 30 fps).

Integration of Equation (3) within the limits of one cycle [0..1/f] (see Appendix A), gives the intrinsic dissipation per loading cycle $d_{1}^{N}$:

(5) $$d_{1}^{N}=\rho C_{\varepsilon ,a}T_{amp}\cdot sin\left(\frac{\phi -\phi_{ref}}{2}\right)$$

with density *ρ*, specific heat capacity *C_ε,α_*, lock-in amplitude of measured Temperature *T_amp_*, and the shift in lock-in phase *ϕ* related to the lock-in phase in the reference area *ϕ_ref._*

In other words, using Equation (5) and the measured lock-in amplitude and phase leads to an estimate of the dissipational energy, which is supplied to the specimen on every loading cycle. The stored energy can also be understood as the available energy to enlarge the cracked surface or to detach the cohesive forces between adjacent laminate-plies, as is explained by the cohesive zone model. Note that in this case a viscoelastic cohesive zone model should be more suitable, as it can account for characteristic time dependent effects in polymer-based matrix composites, e.g., stress-rate, retardation, relaxation and more.

Contemplating again on the fracture mechanical formulation for the idealized situation of mode-I delamination in a double cantilever beam specimen, the crack growth per loading cycle can be expressed by a Paris-law like formulation in the stable region [16].

(6) $$\frac{da}{dn}=c{\left(\frac{G_{i,max}\left(a\right)}{G_{IC}\left(a\right)}\right)}^{m}$$

where *G_i_*_,*max*_ is the maximum occurring strain energy release rate at crack length *a* and *G_IC_* is the critical strain energy release rate at crack length *a*. 

This formulation seems applicable to double cantilever beam specimens, where the crack propagates in a certain direction with a plane crack front and the crack tip velocity being determined by the maximum strain energy release rate. The latter is typically located in the middle of the thumbnail-shaped crack front (e.g., in [15]). In contrast, the delaminations within the hybrid-joint do not grow in a plane crack front, but in any direction that is perpendicular to high stresses. Accordingly, the authors assume that one value—the maximum strain energy release rate—is not representative of the heterogeneous crack growth of the whole specimen. Hence, a more appropriate formula should account for the different strain energy release rates occurring along the specimen-volume. Here, the volume consists of volume-elements each being represented by one pixel of the thermography-image. Considering the sum of Equation (6) for all occurring *G_i_* values and replacing *G_i_* by the dissipation *d*_1_, the incremental growth rate of the delaminated Area *dA*/*dN* follows:

(7) $$\frac{dA}{dN}=c\cdot \underset{m,n}{\sum }{\left(\frac{d_{1,mn}^{N}}{G_{IC}}\right)}^{z}$$

with *c*, *z* being material dependent parameters and $d_{1,mn}^{N}$ are the values of intrinsic dissipation (see Equation (5)) calculated for each Pixel *m*, *n*.

### 3.4. Determination of the Model Parameters

Due to the scattering in lifetime to failure (e.g., Figure 5) and the determined values of the critical strain energy release rate, *G_IC_* is not considered deterministic but normally distributed with a mean value *µ* = 486 J/m^2^ and standard deviation *σ* = 84 J/m^2^.

The determination of the model parameters *c* and *z* (Equation (7)) is executed using the experimental results of the hybrid-joints with PPA-inserts ‘B14’, ‘B16’, ‘B18’, ‘B24’ and ‘B32’. Based on the thermography measurands ‘temperature amplitude’ *T_amp_* and ‘delamination size’ *A*, Equation (8) depicts the analytical expression for the overall Error *Y* to find a suitable set of parameters c and z using the least-squares method. Since the exact values for *G_IC_* of the specimens are not known, Equation (8) is solved numerically with 200 repetitions and normally distributed *G_IC_*. Note that the calculation speed is enhanced, if the dissipation-values *d*_1_ of all pixels are reduced to a 100-bin histogram and the calculation is running on the histogram-data. See Figure 10a,c for exemplary histogram-data of the intrinsic dissipation *d*_1_.

(8) $$Y=\underset{P}{\sum }\underset{I}{\sum }{\left(c\cdot \underset{m,n}{\sum }{\left(\frac{d_{1,mn}^{N}}{G_{IC}}\right)}^{z}-\frac{dA}{dN}\right)}^{2}$$

(9) $$c,z\;\to \min\left(Y\right)$$

where *Y* is the overall Error with set of parameters (*c*, *z*), *P* is the lot of validation samples (‘B14’, ‘B16’, ‘B18’, ‘B24’ and ‘B32’) and *I* is the set of measurement Points.

**Figure 8:**
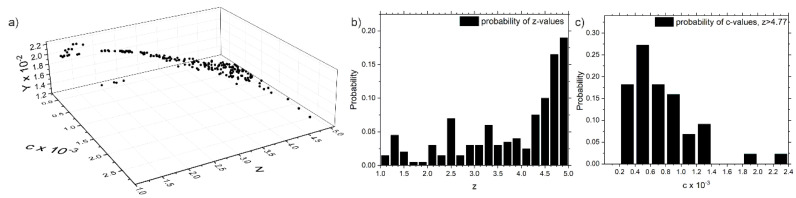
(**a**) Numerically calculated solution space of equation 5.7 for paired values *c*, *z*, resulting in the least Error *Y*, (**b**) 20-bin histogram of *z*-values and (**c**) histogram of *c*-values for *z* lying within the most probable bin (*z* > 4.77).

Thus, 200 solutions for *c* and *z* result, each representing the least difference between the calculation-model (Equations (7) and (8)) and the experimental values. The room of these 200 solutions and the histograms of *z* and *c*-values, respectively, are given in Figure 8, pointing out that all the obtained solutions for *c* and *z* lie within a certain domain. However, *z* results from the histogram-bin with the most probable occurrence being 4.86 (Figure 8b). This histogram-bin, *z*-values exceeding 4.77 leads to a corresponding set of *c*-values, whose mean-value will be taken as model parameter c = 7.46 × 10^−4^ for the latter calculations.

### 3.5. Model Validation

To validate the model and the obtained parameters *c =* 7.46 × 10^−4^ and *z =* 4.86 (Section 3.4), the following section compares the experimentally obtained delamination size with those resulting from the numerical calculations based on Equations (5) and (7). In a first step, the delamination size is calculated for the samples used to identify the model parameters in Section 3.4 (see Figure 10). In a second step, the calculation model is validated by applying it to experimental data sets of the ‘A-series’.

Since the critical strain energy release rate of each specimen is vague, again, *G_IC_* is interpreted as a normal-distributed random value with mean *µ* = 486 J/m^2^ and standard deviation *σ* = 84 J/m^2^. Thus, to achieve a confidential validation, the numerical calculations carry out a loop with 100 repetitions. 

**Figure 9:**
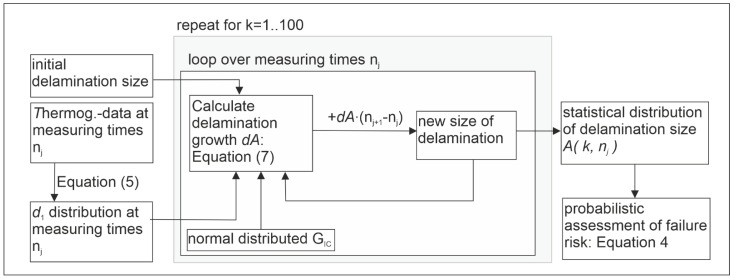
Illustration of the data flow and the calculation routine.

Recalling Equation (6), the calculation routine uses the experimental thermography data (temperature amplitude and phase) to compute the distribution of the intrinsic dissipation *d*_1_, which results in calculating the growth of delamination (Equation (7)). Figure 9 illustrates the calculation routine, which was written as a ‘.m’-script (i.e., Matlab, see Appendix B).

**Figure 10:**
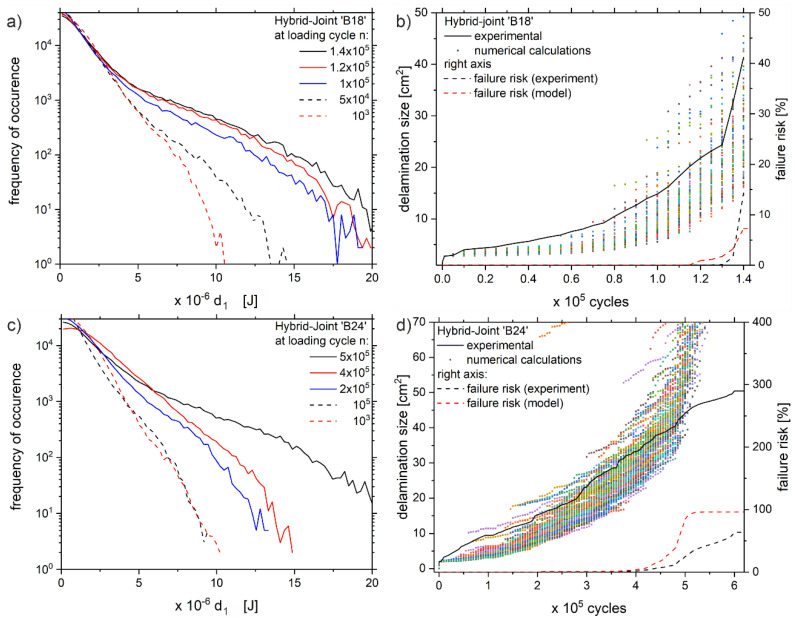
Distribution of dissipation values *d*_1_ for samples (**a**) ‘B18’ and (**c**) ‘B24’ at five different measuring times n, with the corresponding evolution of delamination size during the experiments of (**b**) ‘B18’ and (**d**) ‘B24’, including experimental data (solid line), probabilistic calculated defect size (colored dots) and the resulting failure risk (dashed lines, referring to right axis).

Finally, Figure 10 shows the results for the specimen ‘B18’ and ‘B24’. For both samples, the distribution of *d*_1_ is given at five measuring times (a) and (c), respectively. Secondly, Figure 10b,d compare the real growth of the delamination during the experiment with the calculated probabilistic results.

Note that the calculation starts at loading cycle *n* = 1000. Otherwise, the model slightly underestimates the fast delamination growth within the first 1000 loading cycles. Furthermore, it has to be reminded that for the cross-sectional area of one pixel (0.03875 mm^2^) the interlaminar tensile strength *G_IC_* equals 1.88 × 10^−5^ J. 

It is evident from Figure 10a,c that the center of gravity of the dissipation values lies around 10^−6^ *J*. With increasing amount of loading cycles, the distribution progressively shifts towards higher *d*_1_ values and even reaches above 10^−5^ *J* just before upcoming failure. Hence, the fast delamination growth, i.e., visible for the ‘B18’ specimen from 1.3 × 10^5^ until 1.4 × 10^5^ loading cycles, can mainly be assigned to the occurrence of high *d*_1_ values. In contrast, the dissipation values of the ‘B24’ sample are much lower than those of ‘B18’ and therefore the delaminations grow slower. Furthermore, from Figure 10c,d it seems that the continuous change of the *d*_1_ distribution is directly correlated with the steady delamination growth, which appears in Figure 10d (black solid line).

Now focusing on the evolution of the delamination size (b) and O(d), the experimentally observed size (black solid line) lies very well within the statistical scattering of the probabilistically calculated values. However, the model results in a slight underestimation in the early state and a slight overestimation of the taller delaminations. The latter is most likely due to the higher deformation of the specimen, artificially elevating the temperature amplitude *T_amp_* of the thermography data due to local differences in the emissivity and small out-of-plane deformations. 

Integrating the experimental and the calculated delamination sizes into the Weibull-fit derived from Figure 6 and Equation (4), respectively, results in the failure risk (dashed lines Figure 10). For the ‘B24’ sample, both lines simultaneously start to increase, whereas the calculated failure risk shows a higher slope. Especially above 4.7 × 10^5^ cycles, the faster increase towards 100% is obvious. Again, this can be attributed to the artificially elevated dissipation values *d*_1_. In contrast, the failure risk does not necessarily has to reach high values before the samples break, see ‘B18’. Due to the rapid delamination growth of the ‘B18’ sample between 1.3 × 10^5^ and 1.4⋅10^5^ cycles, the experimental failure risk is negligible until then. According to the probabilistic calculation, at 1.2 × 10^5^ cycles the delamination can reach a size up to 40 cm^2^. Hence, the model related failure risk increases much earlier and seems to be a viable indicator for the upcoming failure.

**Figure 11:**
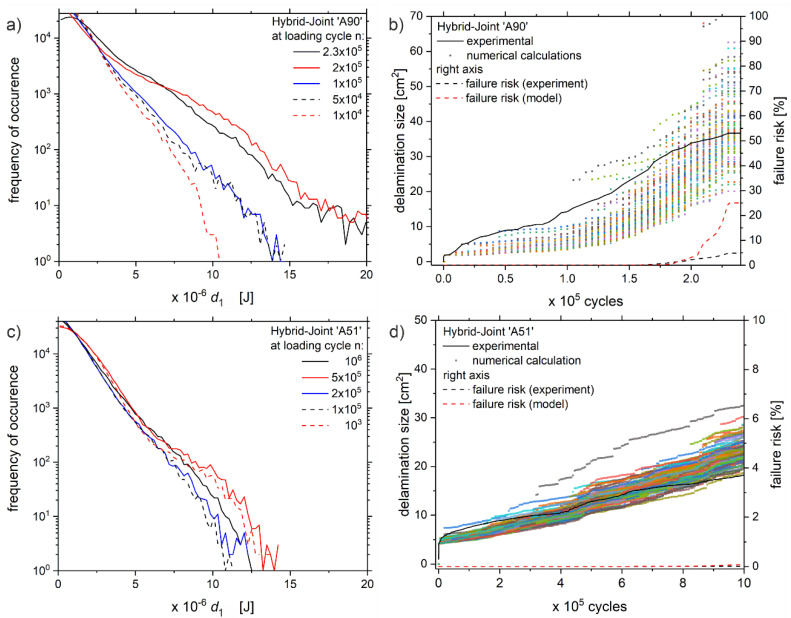
Distribution of dissipation values *d*_1_ for samples (**a**) ‘A90’ and (**c**) ‘A51’ at five different measuring times n, with the corresponding evolution of delamination size during the experiments of (**b**) ‘A90’ and (**d**) ‘A51’, including experimental data (solid line), probabilistic calculated defect size (colored dots) and the resulting failure risk (dashed lines, referring to right axis).

In the second step of the validation, the calculation model is applied to data sets of the ‘A-series’ with the intermittent layer consisting of PPAGF30. These data sets have not been considered in the determination process of the model parameters. Figure 11 compares the results of the calculation model and the experiment.

The results in Figure 11 confirm the observations and interpretations stated previously for the ‘B-series’. Again, the model reproduces the growth of the delamination very well, while a slight underestimation of the delamination size in the beginning and a slight overestimation to the end of the experiment are evident. However, the calculation model fits both, the early failing sample ‘A90’ as well as the runout ‘A51’, even though the intermittent layer consists of PPAGF30 and the model parameters *c*, *z* have been defined for the B-series with PPA. These materials do have different material properties, e.g., E-Modulus, tensile strength, which can have a crucial influence on the fatigue resistance or even the damage mechanisms of the hybrid-joint [26]. Consequently, the model parameters *c, z* are valid for the examined hybrid-joint with PPA and PPAGF30 as the thermoplastic interlayer.

Ultimately, it can be concluded, that the calculation model agrees well with the experimental data, although the experimental results cover a wide range from early rupture to long-lasting specimen. Thereby it needs to be emphasized that the presented approach facilitates a probabilistic calculation of the delamination growth based only on quantitative thermography measurands. 

## 4. Conclusions

This section is not mandatory but can be added to the manuscript if the discussion is based on experimental results on a hybrid-joint, i.e., mechanical fatigue testing with in situ passive thermography, this work elaborates a new approach that incorporates the quantitative analysis of thermography data in a fracture mechanical description for the mode-I delamination growth. The presented probabilistic model facilitates the calculation of the failure risk and gives a probabilistic estimate of the delamination size, which agrees well with the experimental data.

The following conclusions are the basis of the derived model. The fatigue damage of the hybrid joint is dominated by mode-I delamination growth. While the delamination size does not provide information on the crack growth rate, it is a viable indicator for failure risk. Hereby, the Weibull-cumulative distribution function is an appropriate fit of the failure risk. 

Meanwhile, damage is accompanied by thermal effects, which are measured quantitatively. Based on the local heat conduction equation, a new formulation is derived incorporating the lock-in amplitude and phase to calculate the intrinsic dissipation. Subsequently, the presented approach proceeds with the intrinsic dissipation to calculate the incremental growth of the delaminations.

This work executes the presented approach in a probabilistic perspective facilitating the calculation of the failure risk and the delamination size, which agrees well with the experimental data. The validation of the model and parameters shows great accordance with the experimental and the calculated data, even though the experiments cover a range from early rupture to runouts. Thus, the found parameters are valid for the investigated hybrid joint with PPA and PPAGF30.

## Figures and Tables

**Table 1:** Experimentally determined values of crititcal strain energy release rate *G_IC_.*

Specimen	3-1	3-2	3-3	4-1	4-2	4-3
*G_IC_* [J/m^2^]	496.5	435.7	495.9	493.0	538.2	504.6
Mean [J/m^2^]	486.7					
Standard deviation [J/m^2^]	84					

## Data Availability

The data presented in this study are available on request from the corresponding author.

## Appendix A

Underlying theorem for Equation (5):

Temperature change due to thermoelasticity:

$$\Delta T_{el}\left(t\right)=-\frac{aT_{0}\sigma_{a}}{\rho C_{p}}\sin\left(2\pi ft\right)$$

Temperature change due to dissipation:

$$\Delta T_{diss}\left(t\right)=\frac{N}{\rho C_{p}}\int_{0}^{\varepsilon_{p}\left(t\right)}\sigma_{a}\sin\left(2\pi ft\right)d\varepsilon_{p}=\frac{N\varepsilon_{p}\left(t\right)}{\rho C_{p}}\sigma_{a}\sin\left(2\pi ft+\delta \right)=\;T_{a}\sin\left(2\pi ft+\delta \right)\;\#$$

where $T_{a}=\frac{N}{\rho C_{p}}\sigma_{a}\varepsilon_{p}\left(t\right)$ and *δ*: plasticity induced phase shift.

Considering the phase of the thermoelasticity term being 0 and the phase shift *φ* between thermoelastic effect and dissipation as φ=π+δ, the total balance reads:

$$\begin{array}{cc} \Delta T\left(t\right) & =-\frac{N\varepsilon_{p}\left(t\right)}{\rho C_{p}}\sigma_{a}\sin\left(2\pi ft+\delta \right)+\frac{aT_{0}\sigma_{a}}{\rho C_{p}}\sin\left(2\pi ft\right)\\ & =\frac{N\varepsilon_{p}\left(t\right)}{\rho C_{p}}\sigma_{a}\sin\left(2\pi ft+\pi +\delta \right)+\frac{aT_{0}\sigma_{a}}{\rho C_{p}}\sin\left(2\pi ft\right)\\ & =\frac{N\varepsilon_{p}\left(t\right)}{\rho C_{p}}\sigma_{a}\sin\left(2\pi ft+\frac{\varphi }{2}\right)+\frac{aT_{0}\sigma_{a}}{\rho C_{p}}\sin\left(2\pi ft-\frac{\varphi }{2}\right) \end{array}$$

Integration of the time-derivation of *T*(*t)* in the limits of one cycle [0..1/f] results in:

d dt→∮01fdt→ ∮01fT˙dt=∮01fddtNεptρCpσasin2πft+φ2+aT0σaρCpsin2πft−φ2dt=NσaρCpεp1f·sin2π+φ2−εp0·sin0+φ2+aT0σaρCpsin2π−φ2−aT0σaρCpsin0−φ2=NσaρCpεp1f·sinφ2−εp0·sinφ2+0=NσaρCpΔεpNsinφ2

Therefore, $d_{1}^{N}=\rho C_{p}\dot{T}=\rho C_{p}T_{a}\sin\left(\frac{\varphi }{2}\right)$.

## Appendix B

(…)

% Forward Calculation of damage with model paramters c, z

%%

Pixel_size = 0.03875; % [mm2]

Num_rep = 100;

Loading_cycles_per_loop = 5000;

Weibull_fit_a = @ (size_delam) 1-exp (-(size_delam/A) ^ b);

p_fail = zeros (size (sample_name,3), 1);

figure;

plot(delamination_experiment_sample);

title (‘Damage ‘sample_name’-Calculation’); hold

% loop over number of repetitions for statistics

for n = 1:num_rep

%set model parameters

z = 4.86;

c = 7.4624 × 10^−4^;

% starting conditions at n = 11 (1000 loading cycles)

Delam (n, 11) = delamination_experiment_sample (11);

% loop over number of measuring times

for I = 12: size (delamination_experiment_sample, 3)

% get normal distributed value for interlaminar tensile strength

G_ic = prop_distribution (‘gauss’,486.7,84). * 1 × 10^−6^ * pixel_size;

% calculate new delamination size

Delam (n, i) = delam(n, I − 1) + pixel_size * c. * sum(histogram_d1_values_sample(:, i). *…

(histogram_centers_d1_sample (:, 1)./G_ic). ^ (z)). * loading_cycles_per_loop;

% estimate failure risk

p_fail (i) = p_fail (i) + Weibull_fit_a (delam (n, i));

end

plot (abs (delam (n, :)), ‘o’);

end

figure;plot (p_fail./n);
